# Desmoid Tumors Arising on the Mesenteric Surgical Scar of Abdominal Sarcomas

**DOI:** 10.7759/cureus.21727

**Published:** 2022-01-30

**Authors:** Giovanni Damiani, Rabih Mikhael, Dimitri Tzanis, Sophie El Zein, Sylvie Bonvalot

**Affiliations:** 1 Surgery, Institut Curie, Paris Sciences et Lettres (PSL) University, Paris, FRA; 2 Pathology, Institut Curie, Paris Sciences et Lettres (PSL) University, Paris, FRA

**Keywords:** mesenteric tumor, retroperitoneal tumor, sarcoma surgery, soft tissue tumor, oncologic surgery, desmoid tumor

## Abstract

A sporadic desmoid tumor (DT) is a rare type of tumor of the mesenchymal connective tissues is now considered an intermediate disease or locally aggressive. They may develop on scars or after traumatism, favored by growth factors released during the initial phase of wound healing. Most of the abdominal DT arising on a scar is described on the wall incision. In this report, we describe two cases of DT arising on the intraperitoneal surgical scar, shortly after the resection of a low-grade retroperitoneal liposarcoma and a low-risk gastric gastrointestinal stromal tumor (GIST). Inconsistency between low risk according to the classification of the primary sarcoma and early local recurrence (LR) should raise the possibility of DT. Core needle biopsy (CNB) should be performed when it is feasible, including on local recurrences (LR). Surveillance has become the first-line treatment for DT. In case of progression between two imaging during the surveillance phase, surgery, when it's not mutilating, is indicated for selected cases as second-line treatment.

## Introduction

A sporadic desmoid tumor (DT) is a rare type of tumor of the mesenchymal connective tissues classified in the intermediate diseases (locally aggressive) in the 2020 World Health Organization (WHO) classification [1]. The incidence is five to six cases per million individuals per year [2]. They occur in the deep soft tissues everywhere in the body [1]. Five to ten percent arise in the context of familial adenomatous polyposis (FAP). This is a monoclonal fibroblastic proliferation with uniform spindle cells [1]. Approximately 85%-90% of sporadic DF harbors wild-type mutations in the beta-catenin gene. In FAP, preferential location is mesentery, with a constitutional APC mutation [2]. These two mutations are exclusive. Desmoids are known to develop eventually on scars or after a traumatism, and growth factors released during the initial phase of wound healing could transmit signals which promote the activation of beta-catenin [3]. Scar-related abdominal desmoids are almost always on the route of the wall incision, for instance on the previous caesarian sections [4]. In this setting, diagnosis is easily done by percutaneous core needle biopsy (CNB), according to the guidelines [2]. In this paper, we describe two cases of DT arising shortly after the resection of two abdominal sarcomas. These DT were situated on the mesenteric scar of the previous surgery. Here, we discuss how to anticipate this diagnosis.

## Case presentation

Case 1

**Figure 1:**
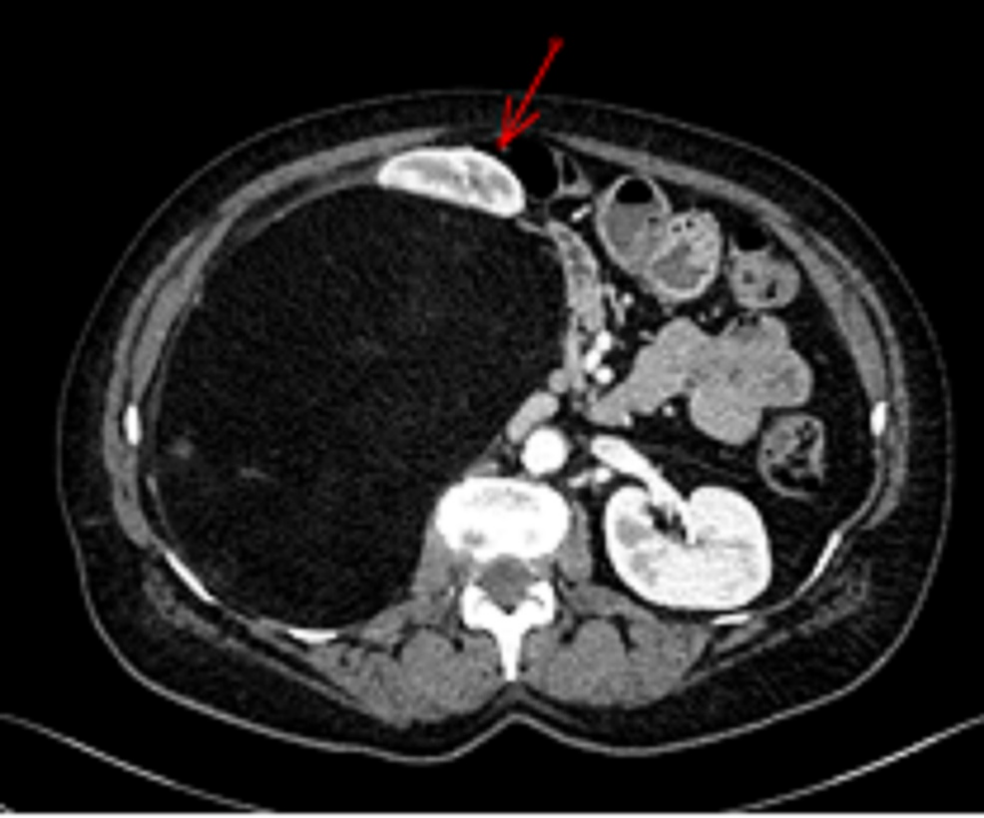
Case 1. Contrast-enhanced abdominal CT scan This shows a retroperitoneal adipocytic mass displacing the right colon and kidney to the left (red arrow).

**Figure 2:**
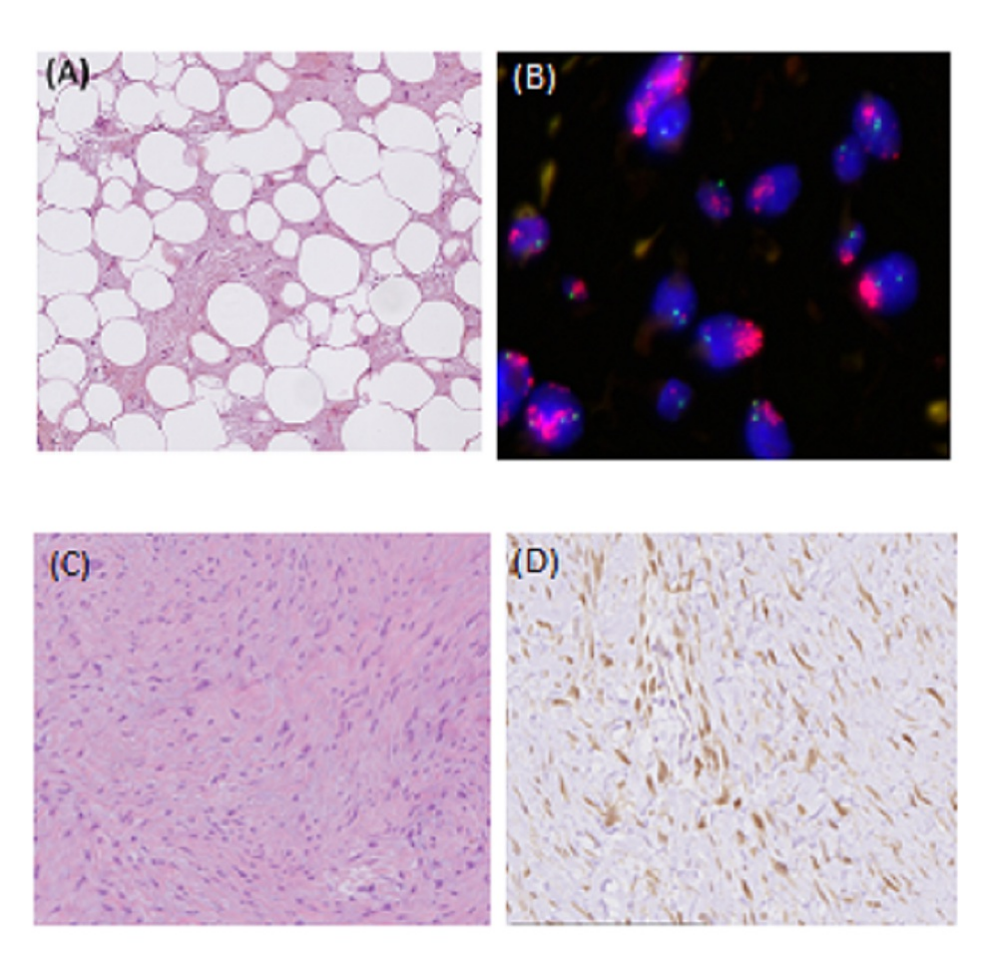
Case 1. Well-differentiated liposarcoma as the primary tumor G stands for magnification (A) Gx10 tumor is composed of mature adipocytes with significant variation in cell size and of atypical hyperchromatic stromal cells admixed within a collagenous background. (B) Interphase fluorescence in situ hybridization using probes for MDM2 (red signal) and the centromere of chromosome 12 (green signal) showed high-level amplification of MDM2 grouped in clusters. MDM2 staining Gx10, the tumor cells showed a moderate nuclear MDM2 staining consistent with a well-differentiated liposarcoma. (C) The Desmoid tumor was made of bland myofibroblasts arranged in long fascicles with a fibrous stroma. (D) β-catenin-staining Gx20 showed a diffuse and strong cytoplasmic and nuclear overexpression, consistent with the diagnosis of DT.

**Figure 3:**
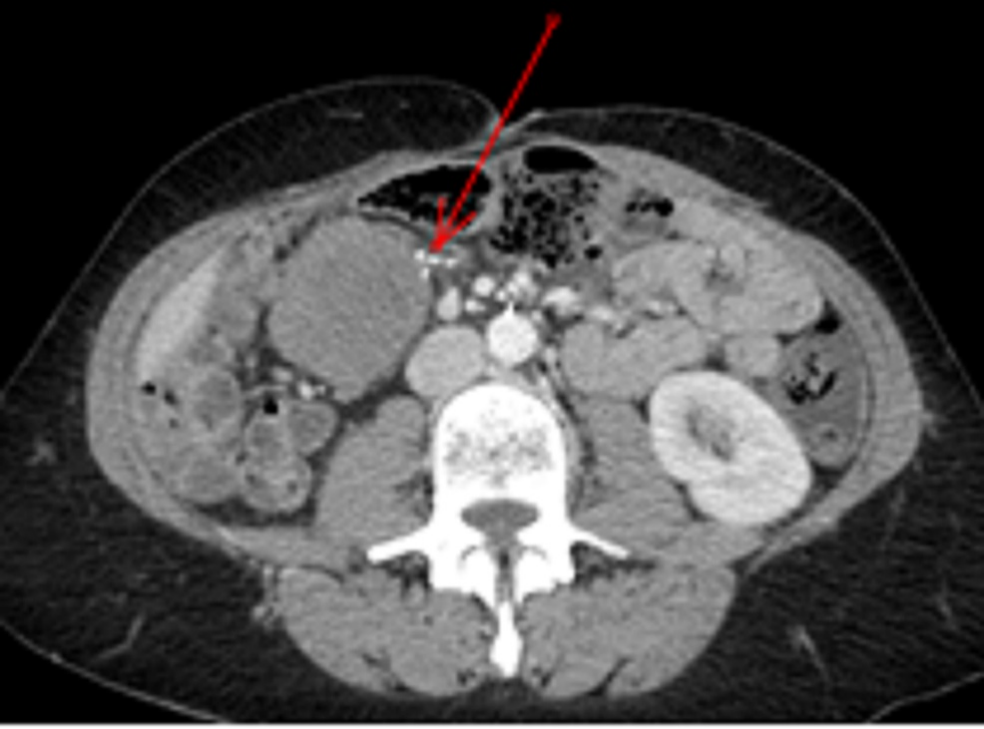
Case 1. Contrast-enhanced abdominal CT scan This shows a well-defined homogenous non-adipocytic round mass in the mesentery close to the staple line of the ileocolic anastomosis (red arrow).

A 50-year-old woman with no specific past history was referred for a retroperitoneal tumor. An abdominal pelvic CT scan showed a homogeneous adipocytic right retroperitoneal mass of 30 cm (Figure 1) encasing the right kidney and pushing the right colon to the left. According to the guidelines, a CNB was performed [5-6]. Pathology revealed a well-differentiated liposarcoma (WDLPS) with amplification of MDM2 on the fluorescence in situ hybridization (FISH) test, which confirmed the diagnosis (Figures 2A-2B). The patient was enrolled in the STRASS protocol, which randomized preoperative radiotherapy versus surgery alone, and was assigned to the surgery alone arm [7]. Surgery consisted of a compartmental resection of the tumor en bloc with the kidney inside, the colon in the front, and the aponeurosis of the psoas in the back [6,8]. The pathological analysis of the specimen confirmed a 30 X 26 cm WDLPS grade 1 with negative margins of resection. Ten months later, the abdominal CT scan revealed a 5-6 cm undifferentiated mass located on the mesentery close to the staple line of the ileocolic anastomosis (Figure 3). Since the location was not accessible to a CNB, the multidisciplinary tumor board (MTB) wrongly considered it a local recurrence (LR). Because of the short interval between the surgery of the primary tumor and the recurrence, the patient received neoadjuvant chemotherapy (CT) with four cycles of doxorubicin [9]. The patient was operated on at the end of the chemotherapy. During surgery, the tumor was attached to the ileocolic anastomosis. The former anastomosis and the mesentery were resected and another anastomosis was performed. The pathology of the specimen revealed a 6 X 5 cm fibroblastic proliferation with no microscopic feature of malignancy (Figures 2C-2D). Fluorescence in situ showed no amplification of MDM2. Genetic analysis showed a mutation in the exon 3 of the CTNNB1 gene (T41A) coding for beta-catenin and confirmed the diagnosis of DT. Seven years later, the patient is disease-free on the last control.

Case 2

**Figure 4:**
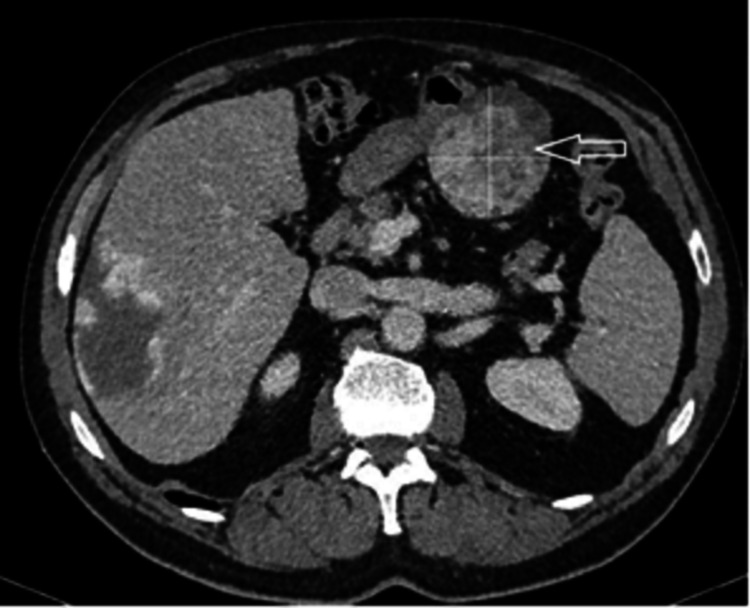
Case 2. Contrast-enhanced abdominal CT scan This shows a heterogeneous mass on the posterior wall of the antrum, part of the stomach (the white cross inside the tumor is to show the borders of the tumor because it is heterogeneous).

**Figure 5:**
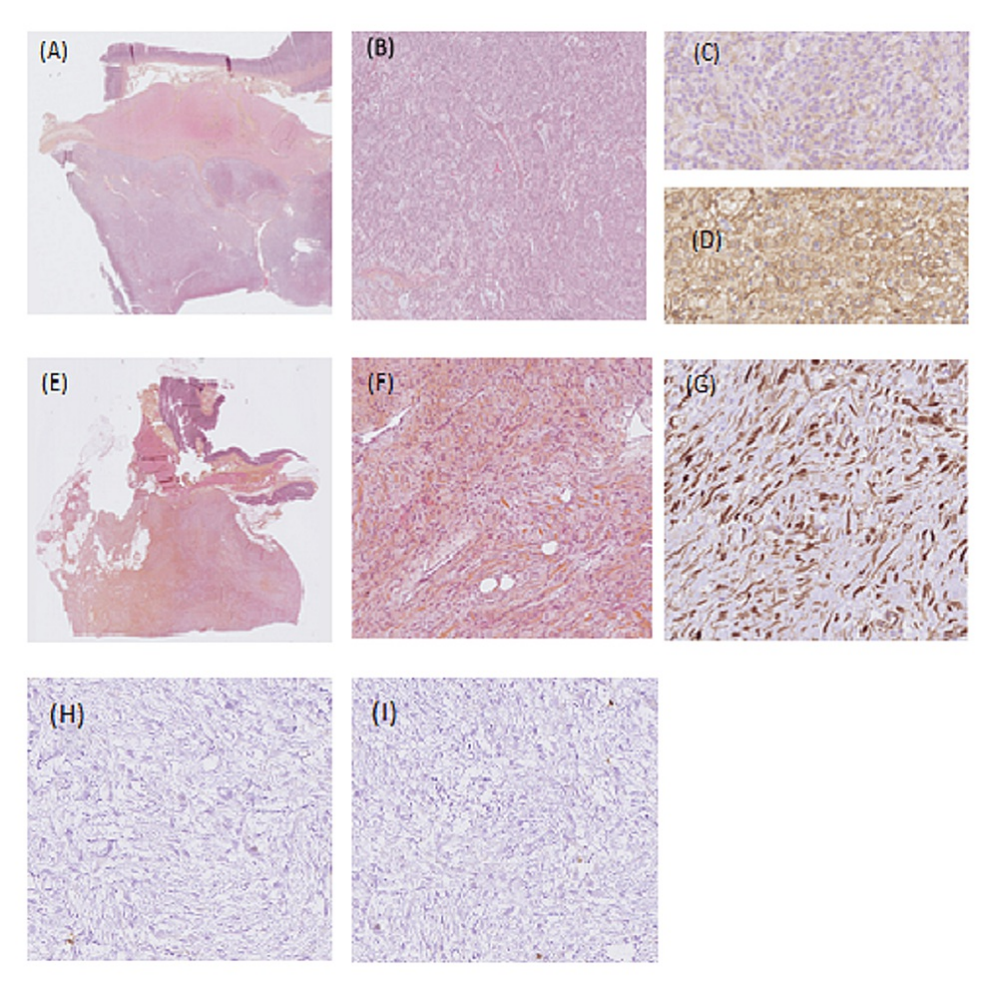
Case 2. GIST epithelioid type harboring a PDGFRA mutation G: stands for magnification; GIST: gastrointestinal stromal tumor (A) Gx 0.46, well-circumscribed mass infiltrated the muscular wall (asterisk) and the subserosa of the stomach. (B) Gx5 tumor was made of spindle and epithelioid cells within a myxoid stroma. (C) CD117 staining, Gx20: PDGFRA_mutant GIST showed a characteristic limited and slight expression of KIT. (D) DOG1 staining, Gx20 diffuse expression of DOG1 confirmed the diagnosis of PDGFRA-mutant GIST. Desmoid tumor (E) Gx0, 35: low-power examination revealed a poorly circumscribed lesion with ill-defined infiltration of the subserosa and the muscular wall (asterisk) of the colon. (F) High-power examination revealed bland myofibroblasts without cytonuclear atypia and without necrosis, dispersed within a fibrous stroma. (G) β-catenin staining Gx20 by immunochemistry, tumor cells showed a diffuse and strong nuclear expression of β-catenin, consistent with the diagnosis of desmoid tumor. (H): C-KIT staining Gx10 by immunochemistry: conversely, to a specimen of the primary, tumor cells do not express anymore C-KIT. (I): DOG1 staining Gx10: Conversely to the specimen of the primary, tumor cells do not express any more DOG1.

**Figure 6:**
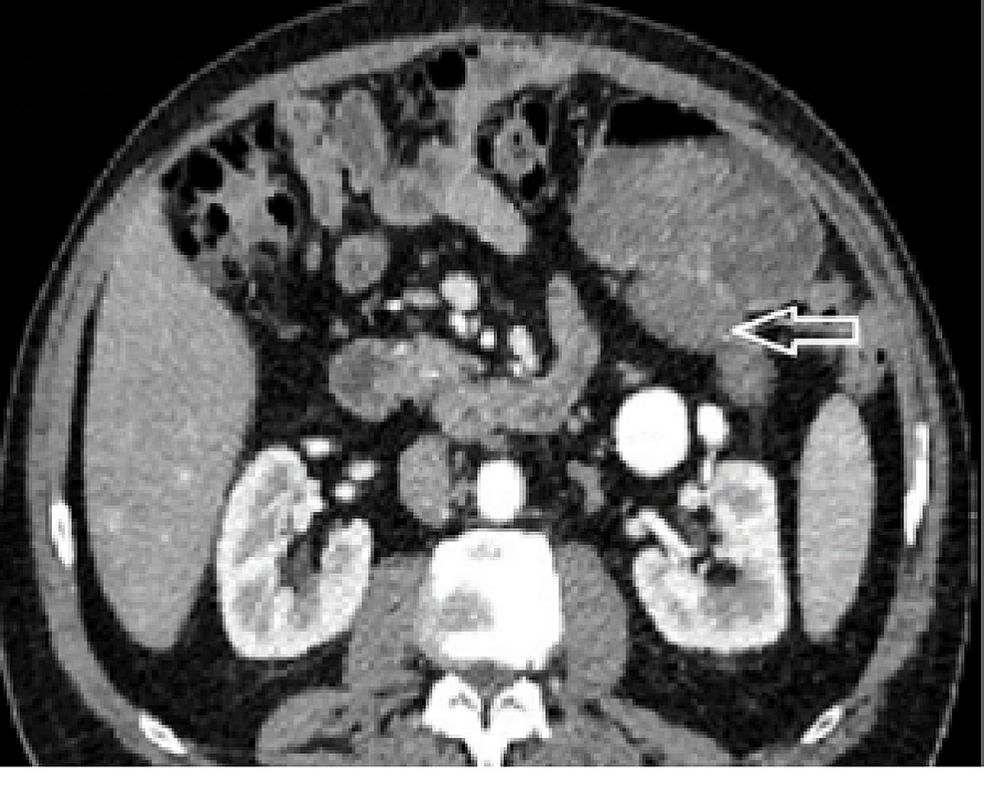
Case 2. Contrast-enhanced abdominal CT scan The white arrow points to a homogenous mass on the posterior wall of the stomach.

A 59-year-old man with abdominal pain had an abdominal CT scan that showed a 60 mm tumor on the gastric wall (Figure 4). An endoscopic ultrasound-guided biopsy showed an epithelial gastrointestinal stromal tumor (GIST) (Figures 5A-5B) with immunohistochemical expression of CD117 (Figure 5C) and DOG1 (Figure 5D). A wedge gastrectomy was realized. Pathology of the specimen confirmed a 7 X 6 cm GIST with 5 mitoses for 5 mm2 classified as low risk of progression according to the Miettinen classification [10]. A mutation D842V on exon 18 of the PDGFRA gene was determined on the molecular analysis (tumors with this mutation are not sensitive to Imatinib therapy but have a low risk of recurrence). According to guidelines, no imatinib adjuvant treatment was undertaken [11]. Four months later, two suspicious nodules appeared in the area of the resection, the largest had a 26 mm diameter. A positron emission tomography (PET) scan did not identify any pathological fixation. Percutaneous and endoscopic biopsies were not feasible. After initial active surveillance, the tumor increased in size up to 39 mm in six months (Figure 6) and the MTB decided on an upfront surgery. At laparotomy, the tumors infiltrated the left mesocolon, the gastric great curve, and the tail of the pancreas in the area of the previous resection. A 2/3 gastrectomy with distal splenopancreatectomy together with a section of the left colonic flexure was performed. On the specimen, the two nodules were 60 x 35 mm and 15 x 9 mm, infiltrating the stomach, colon, and tail of the pancreas. A microscopic study showed a myofibroblastic proliferation with no nuclear atypia and no necrosis (Figures 5E-5F). On immunohistochemistry, there was a nuclear overexpression of beta-catenin (Figure 5G) and no expression of CD117 (Figure 5H) and DOG 1 (Figure 5I), leading to the diagnosis of DT. The confirmation was brought by the molecular analysis, which found a mutation in the exon 3 of the CTNNB1 gene (T41A) coding for beta-catenin. One year later, the patient is disease-free on the last control.

## Discussion

These two cases highlight the fact that DT may arise also on the mesenteric scar of a previous surgery done for abdominal sarcomas. This possibility can be extended to other tumor types. This suggests that discrepancy between initial tumor risk and a short interval with the local recurrence should be managed carefully, and DT is one of the possible diagnoses. This is important to confirm by a biopsy when feasible, since initial management may be active surveillance [2].

Indeed, in both cases, there was inconsistency between the low risk of local recurrence of these two sarcomas and the early event observed. For WDLPS, the risk of local recurrence after a complete surgery is less than 5% and roughly 10% at one and five years [12]. Furthermore, imaging of the recurrence was a dedifferentiated tumor, whereas most of the first local recurrence of WDLPS is also still well-differentiated. GIST is the most common digestive sarcoma and adjuvant therapy with imatinib for three years is the standard treatment for patients with a significant risk of relapse [11]. On the contour maps for estimating the risk of GIST recurrence after surgery, a 7 cm gastric GIST harboring five mitoses for 5 mm² on the microscope, without tumoral rupture has a risk of recurrence lower than 10% at 10 years, the reason why no adjuvant treatment was delivered [13]. The PET scan with 18 FDG identified moderate hypermetabolism of the mesenteric lesion. In both cases, we could have suspected a DT. In the case of primary tumors, the standard of care is to perform a CNB to tailor strategy and surgery [6]. In the consensus approach from the Trans-Atlantic RPS Working Group (TARPSWG), percutaneous CNB confirmation of recurrence is also often useful when possible [9] for a number of reasons: 1) to provide a definitive diagnosis because a variety of other entities can be mistaken for the recurrence of the original primary RPS, for example, DT radiation-associated osteosarcoma or angiosarcoma in the bed of original LPS; 2) to guide the selection of preoperative therapies, including potential targeted therapies; 3) as part of a translational research program or clinical trial; 4) because resection often is challenging and can be morbid and should not be undertaken without due cause. However, in both cases, the recurrence was not accessible to a CNB. Then, the three possibilities were resection, surgical biopsy, or surveillance. The GIST patient was operated on because of tumor progression after an initial period of 6 months of surveillance. The two patients were finally operated on, resection being possible without too much morbidity.

Diagnosis of DT should be confirmed by an expert soft tissue pathologist with a strong recommendation to perform a mutational analysis on the specimens to confirm the diagnosis and guide the workup when appropriate [2]. In our two cases, the final diagnosis of DT on the surgical specimen was confirmed by the molecular analysis: in both cases, there was a mutation of the beta-catenin gene. CTNNB1 and APC mutations being exclusive, routine screening of polyposis is not a standard of care when the DT harbors CTNNB1 mutation [2].

When the DT diagnosis is done before any treatment, active surveillance is now recommended to select patients who do require treatment [2]. The strategy changed a lot during the past 10 years: surgery, which was the cornerstone of the treatment, is no longer the standard in first-line treatment, and surgical indications are limited to selected cases, including progression when a non-mutilating surgery is possible [2,14]. Indeed, large studies demonstrated that more than half of DT cases spontaneously regress or stabilize after initial progression [15], including in abdominal locations [16]. In the GIST, the second case, the patient was placed under surveillance for months because the diagnosis was uncertain. The tumor has progressed, which legitimated its resection [14]. Alternatively, medical treatment could have been an option [2] to avoid multivisceral resection. The tendency to occur on the previous scar is the reason for preferring laparoscopic colectomy to open surgery in patients with familial adenomatous polyposis [17].

Due to the experience gained with these two patients, we managed a new patient, with DT being a possible diagnosis in mind, presented with a circular mass arising around the superior mesenteric artery, which appeared very shortly after a Whipple procedure done for a low-grade neuroendocrine tumor of the head of the pancreas. We decided to perform a biopsy and the pathological diagnosis was also a DT with a CTNNB1 mutation. The patient was placed under active surveillance and is stable without any treatment.

## Conclusions

We presented two patients that were operated on a low-grade/risk sarcoma who developed shortly after the resection a local recurrence. That inconsistency between the low risk, according to the classification of the primary sarcoma, and an early local recurrence should raise the possibility of DT on the previous intraperitoneal scar. Core needle biopsy should be performed whenever it is possible, with molecular analysis, which can help differentiate the diagnosis. Indications of surgery of DT are now limited to selected cases. Active surveillance is now proposed in the first line. Surgical resection, especially when the expected morbidity is low, is discussed in case of disease progression on successive imaging or when the tumor becomes symptomatic.
